# Effect of veliparib (ABT-888) on cardiac repolarization in patients with advanced solid tumors: a randomized, placebo-controlled crossover study

**DOI:** 10.1007/s00280-016-3156-x

**Published:** 2016-10-05

**Authors:** Wijith Munasinghe, Sven Stodtmann, Anthony Tolcher, Emiliano Calvo, Michael Gordon, Mathilde Jalving, Judith de Vos-Geelen, Diane Medina, Dennis Bergau, Silpa Nuthalapati, David Hoffman, Stacie Shepherd, Hao Xiong

**Affiliations:** 1 AbbVie Inc., 1 N Waukegan Rd, North Chicago, IL United States; 2 AbbVie Deutschland GmbH & Co KG, Ludwigshafen am Rhein, Germany; 3 South Texas Accelerated Research Therapeutics, LLC, START, San Antonio, TX United States; 4 South Texas Accelerated Research Therapeutics, LLC, START, START Madrid-Centro Integral Oncológico Clara Campal, Madrid, Spain; 5 Pinnacle Oncology Hematology, Scottsdale, AZ United States; 6 University Medical Center Groningen, Groningen, The Netherlands; 7 Division of Medical Oncology, Department of Internal Medicine, GROW School for Oncology and Developmental Biology, Maastricht University Medical Centre, Maastricht, The Netherlands

**Keywords:** Veliparib, Poly(ADP-ribose) polymerase, PARP inhibitor, QT interval, ECG, Solid tumor

## Abstract

**Purpose:**

Veliparib (ABT-888) is an orally bioavailable potent inhibitor of poly(ADP-ribose) polymerase (PARP)-1 and PARP-2. This phase 1 study evaluated the effect of veliparib on corrected QT interval using Fridericia’s formula (QTcF).

**Methods:**

Eligible patients with advanced solid tumors received single-dose oral veliparib (200 mg or 400 mg) or placebo in a 6-sequence, 3-period crossover design. The primary endpoint was the difference in the mean baseline-adjusted QTcF between 400 mg veliparib and placebo (∆∆QTcF) at six post-dose time points. Absence of clinically relevant QTcF effect was shown if the 95 % upper confidence bound (UCB) for the mean ∆∆QTcF was <10 ms for all time points. An exposure–response analysis was also performed.

**Results:**

Forty-seven patients were enrolled. Maximum mean ∆∆QTcF of veliparib 400 mg was 6.4 ms, with a 95 % UCB of 8.9 ms; for veliparib 200 mg, the maximum mean ∆∆QTcF was 3.6 ms, with a 95 % UCB of 6.1 ms. No patient had a QTcF value >480 ms or change from baseline in QTcF interval >30 ms. Treatment-emergent adverse events (TEAEs) were experienced by 36.2, 48.9, and 47.8 % of patients while receiving veliparib 200 mg, veliparib 400 mg, and placebo, respectively. Most common TEAEs were nausea (12.8 %) and myalgia (8.5 %) after veliparib 200 mg, nausea (8.5 %) and vomiting (8.5 %) after veliparib 400 mg, and nausea (6.5 %) after placebo.

**Conclusions:**

Single-dose veliparib (200 mg or 400 mg) did not result in clinically significant QTc prolongation and was well tolerated in patients with advanced solid tumors.

## Introduction

Tumor cells are commonly deficient in DNA repair processes. The resulting genomic instability not only fosters tumorigenesis, but also provides an opportunity for therapeutic intervention [1]. The poly(ADP-ribose) polymerase (PARP) protein family comprises more than 15 enzymes involved in a variety of cellular processes. PARP-1 and PARP-2 are crucial for single-strand DNA repair through the base excision repair pathway [2]. PARP inhibitors are a class of antineoplastic agents that target PARP-mediated DNA repair pathways, resulting in failure of base excision repair to correct single-strand breaks in DNA [2]. Single-strand breaks eventually lead to double-strand breaks, which are commonly repaired by the error-free homologous recombination (HR) system. However, in tumors with defects in the HR repair machinery (i.e., tumors carrying *BRCA1* and *BRCA2* mutations), repair of the double-strand breaks becomes error-prone, leading to non-viable genetic errors and eventual cell death. PARP inhibitors have shown single-agent activity against solid tumors lacking a functional HR system, and also demonstrate activity in combination with chemotherapy in a number of tumor types [2, 3].

Veliparib (ABT-888) is an orally bioavailable, potent inhibitor of PARP-1 and PARP-2 [4] that delays the repair of chemo- or radiotherapy-induced DNA damage [5–8]. Additionally, veliparib might generate stable PARP-1/2 complexes at the site of DNA damage that may exceed the cytotoxicity of unrepaired single-strand breaks associated with PARP inhibition [9, 10]. In preclinical studies, veliparib was shown to increase the sensitivity of a variety of tumors to temozolomide, cisplatin, carboplatin, and cyclophosphamide, while also demonstrating the ability to cross the blood–brain barrier [4]. In a phase 1 study of patients with *BRCA1/2*-mutated or wild-type tumors, the maximum tolerated dose of single-agent veliparib was 400 mg twice daily [11]. Dose-limiting toxicities at this dose level were grade 3 nausea/vomiting and grade 2 seizure in patients with *BRCA1/2*-mutated and wild-type tumors, respectively. Several phase 3 trials of veliparib are ongoing in patients with solid tumors [12]. Two studies are evaluating veliparib in combination with carboplatin and paclitaxel as the first-line chemotherapy treatment of patients with advanced or metastatic non-small cell lung cancer (NCT02106546, NCT02264990). This combination is also being assessed in patients with human epidermal receptor 2 (HER2)-negative advanced breast cancer carrying deleterious *BRCA1/2* mutations (NCT02163694), and in patients with newly diagnosed advanced ovarian, primary peritoneal, and fallopian tube cancers (NCT02470585). An additional study in patients with early stage triple-negative breast cancer is evaluating the addition of veliparib combined with carboplatin compared to the addition of carboplatin to standard therapy versus standard chemotherapy (NCT02032277). Finally, veliparib is being assessed in combination with temozolomide in patients with newly diagnosed glioblastoma (NCT02152982). Veliparib doses of up to 150 mg twice daily were evaluated in combination with other treatments in those studies.

Preclinical studies in dogs revealed a mild but concentration-dependent increase in corrected QT (QTc) interval by veliparib. However, the clinical relevance of this finding was not clear. International Conference for Harmonisation (ICH) E14 guidance for the clinical evaluation of QT/QTc interval prolongation defines a threshold level for clinically relevant effect as the upper bound of the 95 % confidence interval around the mean effect on QTc of 10 ms (ICH, 2005, 2015) [13, 14]. In this study, the effect of single-dose veliparib administration at doses of 200 mg and 400 mg on cardiac repolarization was compared with placebo in patients with advanced solid tumors.

## Materials and methods

### Study design and patients

This was a multicenter phase 1, single-dose, double-blind, placebo-controlled, randomized, 3-period, 6-sequence crossover study (NCT02009631). The primary objective was to evaluate the effect of veliparib on corrected QT interval using Fridericia’s formula (QTcF) in patients with solid tumors. Secondary objectives were to assess the pharmacokinetics, safety, and tolerability of single-dose veliparib.

Patients ≥18 years of age with a histologically or cytologically confirmed metastatic or unresectable solid tumor for which standard curative measures or other therapy that may have provided clinical benefit did not exist or were no longer effective were eligible. Patients with brain metastases were required to have clinically controlled neurologic symptoms. All patients were required to have an Eastern Cooperative Oncology Group (ECOG) performance score of ≤1 be able to receive oral medication, and have adequate bone marrow, renal, and hepatic function. Exclusion criteria were as follows: QTcF > 470 ms at screening or baseline; electrocardiogram (ECG) abnormalities that would not allow for reliable QTc assessment; inadequate serum potassium, magnesium, or calcium, or free thyroxine (FT4) and thyroid-stimulating hormone outside the normal range, or grade 2 hyponatremia or hypernatremia; history of cardiac conduction abnormalities; history of significant cardiovascular disease; blood pressure and heart rates outside the normal range; history of, or an active medical condition that affected absorption or motility; had received anticancer therapy within the previous 21 days prior to the first dose of study drug or recovered to no better than grade 2 clinically significant adverse event (AE) of the previous therapy; or used drugs with a known risk of QT prolongation and Torsades de Pointes within 7 days before the first study dose. All patients were required to provide their signed informed consent. The study received Independent Ethics Committee and Institutional Review Board approval, and was conducted in compliance with the Declaration of Helsinki and ICH Good Clinical Practice guidelines.

### Treatment

Following screening, on the morning of day 1 of each period, eligible patients received a single dose of veliparib 200 mg, veliparib 400 mg, or placebo according to the treatment sequence assigned at randomization, administered orally between 8.00 and 10.00 am, and approximately 30 min after the start of a low-fat breakfast. Each treatment period lasted 3–7 days, allowing for ≥3 days of drug washout between periods. After study completion, patients were permitted to enter an extension study (NCT02033551) to assess the safety of veliparib as monotherapy or in combination with chemotherapy.

During the study, patients were required to be on stable doses of permitted medications or replacement supplements ≥14 days prior to receiving the first study dose. Vitamins and/or herbal supplements were not permitted unless required for a patient’s medical condition. Patients who were taking replacement supplements such as calcium, magnesium, or potassium were allowed to continue on these supplements. Medications with a known risk of QT prolongation and Torsades de Pointes were not allowed, including serotonin receptor antagonists, due to the potential for cardiovascular interactions, with the exception of palonosetron. Colony-stimulating factor or human erythropoietin use was permitted along with supportive prophylactic drugs unless specifically excluded by the study protocol.

### ECG assessments

Serial, resting 12-lead ECG measurements were performed at screening, on day 1 of each period at 0 h (pre-dose), and at 0.5, 1, 2, 3, 10, and 24 h post-dose, and at the final study visit on day 3 of period 3. ECGs were performed in triplicate except for a single ECG at screening. All ECG tracings were evaluated using the automated signal analysis algorithm of eECG/ABBIOS to measure predefined ECG intervals (RR, PR, QT, and QRS duration). All ECGs were manually verified and adjudicated by an expert over-reader who was blinded to treatment, time, and patient identification. T- and U-wave morphologies were independently reviewed by a qualified cardiologist who was similarly blinded to treatment, time, and patient identification. QTcF was determined as follows:

$$ QT_{C} F = \frac{QT}{{\sqrt[3]{RR}}} $$

A linear mixed effects model was used for the analysis of baseline-adjusted QTcF intervals (∆QTcF) for the first 24 h after dosing with either placebo, veliparib 200 mg, or veliparib 400 mg. The primary endpoint was the baseline-adjusted difference in mean QTcF between the veliparib 400 mg dose and placebo (∆∆QTcF) at six post-dose time points. Absence of QTcF effect was established if the 95 % upper confidence bound for the mean ∆∆QTcF between veliparib 400 mg and placebo was below the 10 ms threshold of clinical significance for all post-dose time points, in accordance with ICH E14 guidelines [13, 14]. For the QT/QTc, PR, and QRS interval variables, appropriate cutoff points defining the categories of the largest change from baseline were determined. For QTc, the cutoff points for these categories were as specified in the ICH E14 guidelines. The dataset for ECG evaluations included all available patients’ data.

### Pharmacokinetic assessments

In each period, blood samples were collected for pharmacokinetic analysis at 0 h (pre-dose), and at 0.5, 1, 2, 3, 10, and 24 h post-dose. Plasma concentrations of veliparib were determined using a validated online solid-phase extraction followed by liquid chromatography with tandem mass spectrometric detection with a lower limit of quantitation for veliparib of 1.05 ng/mL. Veliparib pharmacokinetic parameters were estimated using non-compartmental methods.

### Exposure–response model

To establish the nonsignificance of a hysteresis effect, the criteria discussed in Darpo et al. [15] were applied. Subsequently, linear mixed effects models were tested, with or without an additional lagged effect compartment, depending on the outcome of the test for hysteresis. A final model was chosen based on goodness of fit, plausibility of the parameters, and Bayesian information criterion (BIC). The relationship between ∆∆QTcF and veliparib concentration was modeled using the following equation:

$$ \Delta \Delta QTcF_{i,j} = \eta_{i}^{(2)} + ({\text{Slope}}_{0} + \eta_{i}^{(1)}) \times {\text{Concentration}}_{i,j} + \varepsilon_{i,j} $$

for subject *i* and time point *j*. *η* ^(2)^ represents the inter-subject variability on intercept (fixed effect on intercept is set to zero). Slope_0_ represents fixed effect on slope. *η* ^(1)^ represents the inter-subject variability of the Slope. *ε*_*i,j*_ is the residual variability in subject *i* at time *j*.

### Safety

Safety was evaluated by AE monitoring, vital signs, physical examination, and laboratory testing throughout the study. Treatment-emergent AEs (TEAEs) were recorded from the time of first dose of study drug until 30 days from the last day of dosing or discontinuation, or until the first dose of study drug for patients enrolling in the NCT02033551 extension study. TEAEs were classified by Medical Dictionary for Regulatory Activities version 17.0 system organ class and preferred term. The severity of AEs was rated according to the National Cancer Institute Common Terminology Criteria for Adverse Events version 4.0. The safety population consisted of all patients’ data.

### Sample size calculation

For the sample size calculation, it was assumed that veliparib 400 mg would be associated with mean QTcF values higher than placebo by 2–4 ms at each of the six post-dose time points. The standard deviation (SD) of QTcF in oncology patients was assumed to be 20 ms. Complete data from 36 patients would yield 85 % power to demonstrate that the veliparib 400 mg regimen compared to placebo would have no clinically relevant effect on QTc interval (i.e., the 95 % upper confidence bound for the mean ∆∆QTcF would be <10 ms for all six post-dose time points). The target sample size was 48 patients, to allow for potential premature discontinuations and to account for missing or unevaluable ECG measurements.

## Results

### Patient disposition and demographics

Forty-seven patients were enrolled and randomized to the sequence groups shown in Table 1. Patient demographics and baseline characteristics are summarized in Table 1. Patients had a median age of 58.0 years (range, 34–80), with most patients aged <65 years (72.3 %). The majority of patients were female (85.1 %) and white (95.7 %). One patient prematurely discontinued after receiving the veliparib 200 mg–veliparib 400 mg sequence, and not placebo, due to AEs considered by the investigator to have no relationship to study drug.

**Table 1:** Patient demographics and baseline characteristics

Parameter	Regimen sequence^a^						Total *N* = 47
	ABC^b^ *N* = 8	CAB *N* = 8	BCA *N* = 8	BAC *N* = 8	ACB *N* = 7	CBA *N* = 8	
Female, *n* (%)	7 (87.5)	7 (87.5)	6 (75.0)	7 (87.5)	7 (100)	6 (75.0)	40 (85.1)
Race, *n* (%)							
White	8 (100)	8 (100)	8 (100)	8 (100)	7 (100)	6 (75.0)	45 (95.7)
Black or African-American	0	0	0	0	0	1 (12.5)	1 (2.1)
Asian	0	0	0	0	0	1 (12.5)	1 (2.1)
Age, years							
<65, *n* (%)	6 (75.0)	5 (62.5)	6 (75.0)	6 (75.0)	4 (57.1)	7 (87.5)	34 (72.3)
≥65, *n* (%)	2 (25.0)	3 (37.5)	2 (25.0)	2 (25.0)	3 (42.9)	1 (12.5)	13 (27.7)
Mean (SD)	58.8 (11.0)	60.8 (10.0)	56.0 (11.0)	59.3 (6.5)	53.6 (14.1)	53.5 (13.3)	57.0 (10.9)
Median (range)	58.0 (46–80)	62.0 (48–73)	51.5 (45–71)	60.0 (52–69)	58.0 (34–69)	52.5 (39–80)	58.0 (34–80)
Primary cancer, *n*							
Ovarian	6	5	3	7	3	2	26 (55.3)
Breast	1	1	0	0	2	4	8 (17)
Other	1	2	5	1	2	2	13 (27.7)
ECOG, *n* (%)							
0	5	6	7	4	4	3	29 (61.7)
1	3	2	1	4	3	5	18 (38.3)

*A* single-dose veliparib 200 mg, *B* single-dose veliparib 400 mg, *C* placebo, *ECOG* Eastern Cooperative Oncology Group, *SD* standard deviation

^a^For each regimen sequence: the letter in the first position denotes the study drug administered in period 1, day 1; the letter in the second position denotes the study drug administered in period 2, day 1; the letter in the third position denotes the study drug administered in period 3, day 1

^b^One patient was prematurely discontinued in period 3

### ECG

**Fig. 1:**
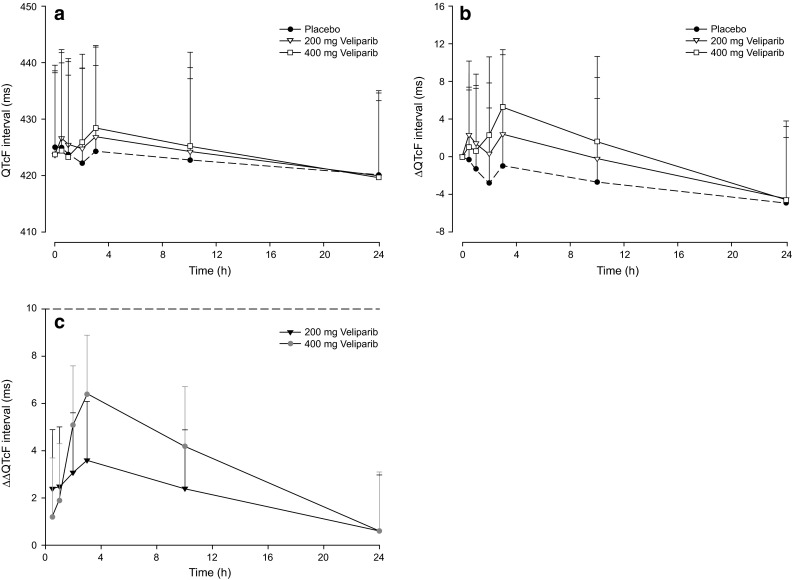
**a** QTcF and **b** baseline-adjusted QTcF (∆QTcF)^a^ interval-time profiles. Data are means + standard deviations. **c** Baseline-adjusted drug-placebo difference in QTcF interval (∆∆QTcF). Data are point estimates plus 95 % upper-bound time profiles. ^a^Four patients had missing baseline or all post-baseline ECG interval measurements and were not included in ∆QTcF analyses for the regimen in which the information was missing. *ECG* electrocardiogram, *QTcF* corrected QT interval using Fridericia’s formula

The mean (+SD) QTcF and mean (+SD) ∆QTcF interval-versus-time profiles on day 1 for veliparib 400 mg, veliparib 200 mg, and placebo are shown in Fig. 1a and b, respectively. No patient while receiving any of the three regimens had a QTcF value greater than 480 ms, and no patient had a change from baseline in QTcF interval greater than 30 ms. Following a single dose of veliparib 400 mg, the maximum 95 % upper confidence bound for mean ∆∆QTcF was 8.9 ms, with estimates of the mean drug effect ranging from 0.6 ms to 6.4 ms at different post-dose time points. For veliparib 200 mg, the maximum 95 % upper confidence bound for mean ∆∆QTcF was 6.1 ms, with estimates of mean drug effect at different post-dose time points ranging from 0.6 to 3.6 ms (Fig. 1c). Therefore, a lack of effect on QTcF interval prolongation was demonstrated for the clinical doses of veliparib [13].

ECG measurements in at least one time point were missing for 13 patients, with some patients missing all ECG replicates at a specific time point. However, since the missing data were dispersed throughout the dosing regimens and assessment time points, their effect on the conclusions was negligible.

### Pharmacokinetics

**Fig. 2:**
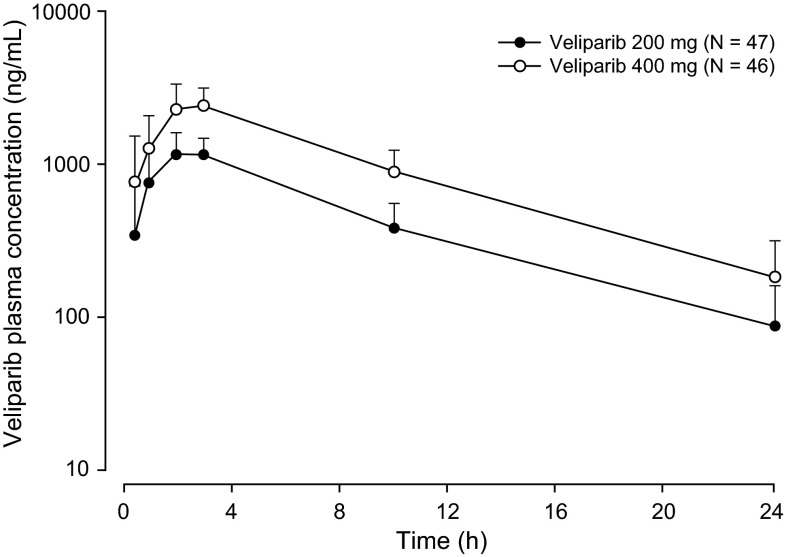
Veliparib plasma concentration–time profile. Data are means + standard deviations; linear-log scale

Following single-dose oral administration of veliparib 200 mg and veliparib 400 mg, systemic exposure to the drug increased in a dose-proportional manner, suggesting a linear pharmacokinetic profile. Consistent with this observation, the dose-normalized maximum concentration (C_max_/dose) and area under the plasma concentration–time curve from time 0 to infinity (AUC_∞_/dose) values were similar between the two dose groups of veliparib (Table 2). Mean time to C_max_ (T_max_) and terminal phase elimination half-life (t_1/2_) were similar for the two dose groups at approximately 2.5 and 5.5 h, respectively. The mean plasma concentration-versus-time profiles for veliparib 200 mg and veliparib 400 mg are shown in Fig. 2.

**Table 2:** Pharmacokinetic parameters of veliparib following single-dose oral administration

Pharmacokinetic parameter	Units	Veliparib	
		200 mg	400 mg
		*N* = 47	*N* = 46^a^
C_max_	µg/mL	1.32 ± 0.378	2.61 ± 0.847
T_max_	h	2.4 ± 0.7	2.5 ± 0.6
AUC_t_	µg h/mL	11.1 ± 3.12	23.8 ± 7.1
AUC_∞_	µg h/mL	12.0 ± 3.98^b^	25.5 ± 8.37
${\text{t}_{1/2}}^ {\text{{c}}}$	h	5.3 ± 1.1^b^	5.4 ± 1.3
CL/F	L/h	18.2 ± 5.09^b^	17.3 ± 5.27
V_Z_/F	L	141 ± 42.8^b^	138 ± 48.4
C_max_/dose	(ng/mL)/mg	6.62 ± 1.89^b^	6.51 ± 2.12
AUC_∞_/dose	(ng h/mL)/mg	59.9 ± 19.9^b^	63.8 ± 20.9

Data are mean ± SD

*AUC*_*∞*_ area under the plasma concentration–time curve from time 0 to infinity, *AUC*_*∞*_ */dose* dose-normalized AUC_∞_, *AUC*_*t*_ area under the plasma concentration–time curve from time 0 to time of the last measurable concentration, *CL/F* apparent oral clearance, *C*_*max*_ maximum observed concentration, *C*_*max*_ */dose* dose-normalized C_max_, *T*_*max*_ time to C_max_, *SD* standard deviation, *t*_*1/2*_ terminal phase elimination half-life, *V*_*Z*_ */F* apparent volume of distribution

^a^One patient experienced vomiting following veliparib administration and exhibited maximum concentrations 10 h post-dose and lower exposure by 1 order of magnitude compared to the rest of the patients in the 400 mg dose group; this patient was excluded from pharmacokinetic parameter summary statistics

^b^ *N* = 46

^c^Harmonic mean ± pseudo SD

### Exposure–response analysis

**Fig. 3:**
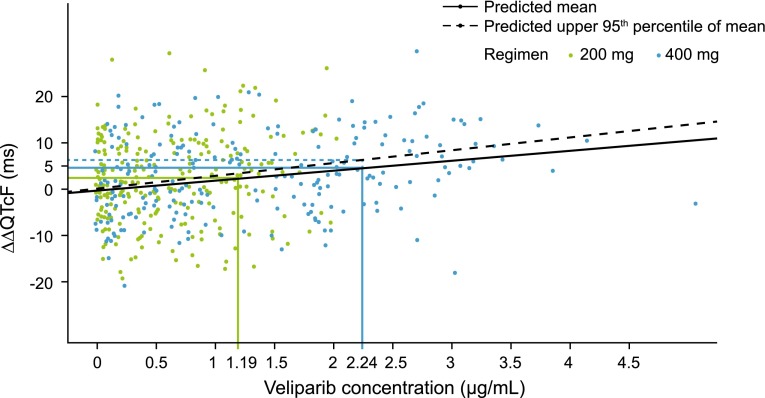
Exposure–response model. Veliparib plasma concentration-∆∆QTcF predictions; data represent median and upper 95 % confidence intervals. *QTcF* corrected QT interval using Fridericia’s formula, *∆∆QTcF* baseline-adjusted drug-placebo difference in QTcF interval

For the exposure–response model, hysteresis was ruled out using the criteria suggested by Darpo et al. [15]. A linear mixed effects model with mean intercept fixed to zero, linking the observed concentration to ∆∆QTcF was found to give the best fit based on BIC. The model showed adequate goodness of fit, considering the variability of the data. The predicted mean and upper 95th percentile ∆∆QTcF at geometric mean of C_max_ in the 200 mg dosing were 2.45 and 3.28 ms, respectively. For the 400 mg dosing, mean and upper 95th percentile ∆∆QTcF at geometric mean of C_max_ were 4.62 and 6.19 ms, respectively (Fig. 3).

### Safety

A similar percentage of patients experienced a TEAE during treatment with veliparib 200 mg, veliparib 400 mg, and placebo (Table 3). More patients who were administered veliparib 400 mg had a TEAE considered by the investigator to be at least possibly related to study drug when compared to either veliparib 200 mg or placebo. The most common TEAEs reported in ≥2 patients in any dosing regimen were nausea (12.8 %) and myalgia (8.5 %) in the veliparib 200 mg dose group, and nausea (8.5 %), vomiting (8.5 %), diarrhea (6.4 %), fatigue (6.4 %), and dizziness (6.4 %) in the veliparib 400 mg dose group (Table 4). In the placebo group, nausea (6.5 %) was the most common TEAE. Overall, nausea was the most common TEAE assessed as having a reasonable possibility of relationship to study drug.

**Table 3:** Treatment-emergent adverse events (safety analysis dataset)

TEAE, *n* (%)	Dosing regimen			Overall
	Veliparib	Veliparib	Placebo	
	200 mg	400 mg		
	*N* = 47	*N* = 47	*N* = 46	*N* = 47
Any AE	17 (36.2)	23 (48.9)	22 (47.8)	38 (80.9)
At least possibly related to study drug^a^	7 (14.9)	11 (23.4)	7 (15.2)	20 (42.6)
NCI CTCAE grade 3 or 4	1 (2.1)	1 (2.1)	0	2 (4.3)
Any serious AE	0	0	0	0
AE leading to discontinuation	0	1 (2.1)	0	1 (2.1)
Any fatal AE	0	0	0	0
Deaths^b^	0	0	0	0

*NCI CTCAE* National Cancer Institute Common Terminology Criteria for Adverse Events, *TEAE* treatment-emergent adverse event

^a^As assessed by investigator

^b^Includes non-treatment-emergent deaths

**Table 4:** Treatment-emergent adverse events reported in ≥2 patients in any dosing regimen (safety analysis dataset)

System organ class Preferred term, *n* (%)	Dosing regimen			Overall
	Veliparib	Veliparib	Placebo	
	200 mg	400 mg		
	*N* = 47	*N* = 47	*N* = 46	*N* = 47
*Gastrointestinal disorders*				
Abdominal pain	2 (4.3)	2 (4.3)	0	3 (6.4)
Constipation	0	1 (2.1)	2 (4.3)	3 (6.4)
Diarrhea	1 (2.1)	3 (6.4)	0	3 (6.4)
Nausea	6 (12.8)	4 (8.5)	3 (6.5)	11 (23.4)
Vomiting	2 (4.3)	4 (8.5)	0	6 (12.8)
*General disorders and administration site conditions*				
Fatigue	2 (4.3)	3 (6.4)	2 (4.3)	7 (14.9)
*Metabolism and nutrition disorders*				
Decreased appetite	0	1 (2.1)	2 (4.3)	3 (6.4)
Dehydration	2 (4.3)	0	0	2 (4.3)
Hypomagnesemia	0	0	2 (4.3)	2 (4.3)
*Musculoskeletal and connective tissue disorders*				
Back pain	0	2 (4.3)	0	2 (4.3)
Myalgia	4 (8.5)	1 (2.1)	1 (2.2)	5 (10.6)
Neck pain	0	2 (4.3)	0	2 (4.3)
*Nervous system disorders*				
Dizziness	0	3 (6.4)	1 (2.2)	4 (8.5)
Dysgeusia	0	2 (4.3)	0	2 (4.3)

One patient discontinued the treatment sequence prematurely after receiving veliparib 200 mg in period 1 and veliparib 400 mg in period 2, due to AEs of suicidal ideation and depression. The patient had a history of depression and had discontinued sertraline, an excluded medication, prior to the first period. Upon resuming sertraline, the events resolved. Both events were considered by the investigator to have no reasonable possibility of relationship to study drug. Two patients experienced a grade 3 TEAE, one each during treatment with veliparib 200-mg and veliparib 400-mg. These events were vomiting and depression, and were considered by the investigator to have no relationship to study drug. No serious AEs or deaths were reported. No safety concerns were observed for any vital sign or biochemical parameter.

## Discussion

Maintaining cardiac function in patients undergoing chemo- and radiotherapy is a concern in the development of any new drug. Advancements in molecular medicine have provided numerous rational targets for therapy, but in some cases, novel treatments such as tyrosine kinase inhibitors have shown the potential to interfere with cardiac repolarization and may therefore present an unacceptable risk to patients who are undergoing cancer treatment [16]. This QT study found no clinically relevant effect of clinical single doses of veliparib (200 mg and 400 mg) on QTcF prolongation per ICH E14 guideline [13, 14]. Consistently, the exposure–response analysis indicated lack of clinically relevant QT prolongation at observed plasma concentration of veliparib with a therapeutic dose.

In this study, patients receiving single-dose veliparib achieved systemic exposure levels to the drug that were comparable with those observed in previous studies, as shown by values for dose-normalized C_max_ and AUC_∞_ [17]. Furthermore, the present findings show that single-dose veliparib at either the 200-mg or 400-mg dose is safe and well tolerated in patients with relapsed/refractory solid tumors.

Originally adopted in 2005 [13], the ICH E14 guidance on evaluating QT/QTc prolongation was deliberately not overly prescriptive, instead focusing on the need during drug development to conduct a “thorough QT/QTc study” [18]. With advances in both science and experience, the ICH has continued to issue updated guidance to overcome ambiguity and uncertainty in relation to the process, with its most recent update in 2015 [14]. In the development of anticancer agents to treat patients with advanced refractory cancer, deviations from the formal ICH E14 guideline have been accepted when the standard QT/QTc study is not feasible for safety or ethical reasons [19]. An alternative approach was used regarding two aspects of study design. First, the study was carried out in the absence of a positive control (such as moxifloxacin) for ethical reasons, to allow an advanced cancer population with no treatment alternative the opportunity to receive a potentially beneficial new cancer therapy without much delay. Second, the highest dose of study drug used was the veliparib 400 mg dose, which might not represent supra-therapeutic exposure. Veliparib 400 mg was chosen in this study for safety reasons, since it was determined to be the maximum tolerated dose of veliparib as single agent in the Puhalla et al. study [11]. Of note, the dose of veliparib used in the ongoing phase 2 and 3 studies when in combination with chemotherapy ranges between 40 mg and 200 mg twice daily. Other than those two aspects, this study was designed and executed with standard attributes of a thorough QT study. Furthermore, the present study represents a successful example of a QT study performed in an oncologic patient population.

The data presented herein support the conclusion that veliparib does not result in clinically relevant QTc prolongation in patients with advanced solid tumors. With an adequate sample size of 47 patients, the data had high precision in establishing the 95 % upper confidence bounds for mean ∆∆QTcF below the threshold of regulatory concern at all post-dose time points. Moreover, there was no safety signal relating to abnormal cardiac repolarization based on the observed AEs, and the drug exposure levels achieved were consistent with previous reports of veliparib in patients with solid tumors.
